# Eco-evolutionary responses to plasmid-dependent phage constrain the spread of multidrug-resistance plasmids

**DOI:** 10.1093/ismejo/wrag113

**Published:** 2026-05-08

**Authors:** Daniel Cazares, Eliza Rayner, Adrian Cazares, Wendy Figueroa, David Goulding, Samuel T E Greenrod, Adam J Mulkern, Michelle Y Y Yin, Tao He, Nick Thomson, Michael A Brockhurst, R Craig MacLean

**Affiliations:** Department of Biology, University of Oxford, Life and Mind Building, South Parks Road, Oxford, Oxfordshire OX1 3EL, United Kingdom; Department of Biology, University of Oxford, Life and Mind Building, South Parks Road, Oxford, Oxfordshire OX1 3EL, United Kingdom; Parasites and Microbes Programme, Wellcome Sanger Institute, Hinxton CB10 1SA, United Kingdom; Parasites and Microbes Programme, Wellcome Sanger Institute, Hinxton CB10 1SA, United Kingdom; Department of Medicine, University of Cambridge, Cambridge CB2 0BB, United Kingdom; Parasites and Microbes Programme, Wellcome Sanger Institute, Hinxton CB10 1SA, United Kingdom; Department of Biology, University of Oxford, Life and Mind Building, South Parks Road, Oxford, Oxfordshire OX1 3EL, United Kingdom; Department of Biology, University of Oxford, Life and Mind Building, South Parks Road, Oxford, Oxfordshire OX1 3EL, United Kingdom; Department of Biology, University of Oxford, Life and Mind Building, South Parks Road, Oxford, Oxfordshire OX1 3EL, United Kingdom; Jiangsu Academy of Agricultural Sciences, Nanjing 210040, China; Parasites and Microbes Programme, Wellcome Sanger Institute, Hinxton CB10 1SA, United Kingdom; Department of Pathogen Molecular Biology, Faculty of Infectious and Tropical Diseases, London School of Hygiene and Tropical Medicine, Keppel Street, London WC1E 7HT, United Kingdom; Division of Evolution, Infection and Genomics, Faculty of Biology, Medicine and Health, University of Manchester, Manchester M13 9NT, United Kingdom; Department of Biology, University of Oxford, Life and Mind Building, South Parks Road, Oxford, Oxfordshire OX1 3EL, United Kingdom; All Souls College, High Street, Oxford OX1 4AL, United Kingdom

**Keywords:** plasmid-dependent phage, plasmid, conjugation, conjugative pili, multidrug resistance, phage resistance, phage therapy, plasmid evolution

## Abstract

Phage therapy offers an alternative to antibiotics for treating multidrug-resistant infections. Plasmid-dependent phages (PDPs) are promising therapeutics as they can kill targeted pathogens and prevent the spread of plasmid-encoded antibiotic resistance genes. However, the evolutionary trajectories of multidrug-resistance (MDR) plasmids under the selective pressure of PDPs remain poorly understood, particularly in eco-evolutionary contexts that remain permissive to plasmid conjugation. We experimentally evolved populations of *Escherichia coli* carrying the MDR plasmid RP4 in the presence of the plasmid-dependent phage PRD1 under conditions where the benefits of conjugation were either strong or weak. When opportunities for conjugation were rare, PRD1 only transiently suppressed the conjugative plasmid population due to the rapid evolution of phage-resistant plasmids lacking conjugative ability. Increasing ecological opportunities for conjugation enhanced plasmid suppression and delayed the evolution of phage-resistant plasmids. PRD1 resistance was associated with plasmid loss and reduced conjugative ability, though this trade-off was complex because resistance mutations caused heterogeneous effects on pilus production and conjugation. Mutations and insertion sequence (IS)-mediated inactivation in conjugation genes generated a spectrum of resistance phenotypes, from partial (*trbB*, *trbL*) to complete (*virB4/trbE*) resistance. Bioinformatic analysis of publicly available IncP plasmids revealed frequent truncations of the VirB4/TrbE protein, suggesting that plasmid-dependent phages may represent an important selective pressure shaping plasmid evolution in natural populations. Our results demonstrate an evolutionary trade-off between conjugative ability and phage resistance that plasmids cannot easily circumvent. Targeting multidrug-resistance plasmids with PDPs is likely to drive loss of conjugation, limiting the transfer of antibiotic resistance genes in microbial communities.

## Introduction

The acquisition of conjugative plasmids carrying dedicated antibiotic resistance genes has played a key role in the evolution of antibiotic resistance in pathogenic bacteria [1, 2]. Plasmids carrying antimicrobial resistance (AMR) genes tend to be associated with small fitness costs [3], and conjugation enhances the ability of plasmids to persist in the absence of antibiotic use [4–6], especially in communities [7–10]. Given the inherent stability of plasmids [11–14], it is important to develop active interventions to combat plasmid-mediated AMR [6, 15–18]. This approach is likely to be particularly relevant for AMR plasmids that are distributed across connected bacterial host species and ecological niches (i.e. human, environment, agriculture) as interventions that target only one niche or host species may have limited impact on the prevalence of the plasmid [19–21].

Plasmid-dependent phages (PDPs) use the conjugative pilus of plasmids as a receptor to infect bacterial cells [22–27]. PDPs are able to infect a wide range of bacterial hosts [28], making them a promising tool to combat AMR plasmids that are widely disseminated across bacterial strains and/or species [29, 30] in bacterial communities that represent important reservoirs of antibiotic resistance, such as the gut microbiome [22, 31, 32]. A key challenge of using phage to combat AMR is that resistance can rapidly evolve, potentially compromising the efficacy of phage treatment [33]. For example, plasmids can adapt to PDPs through mutations that lead to a loss of the ability for conjugation [32]. In the short term, the loss of conjugative genes is likely to lead to a decreased ability of AMR plasmids to spread and persist in microbial communities, and the loss of conjugative ability is associated with an increased extinction probability of plasmid lineages over the long term [34].

Evolutionary responses to PDPs have been studied by exposing monocultures of plasmid-carrying bacteria to phage. However, the lack of potential plasmid recipients in these experiments creates a scenario where conjugative ability is associated with very weak benefits [35, 36]. Metabolic costs of conjugation often drive the evolution of non-conjugative plasmids under these conditions, even in the absence of phage pressure [37–39]. Given that trade-offs are likely to exist between conjugative ability and PDP resistance, we reasoned that increasing the potential for conjugative plasmid transfer—by creating conditions favourable for pilus production—might reduce the strength of selection for PDP resistance and enhance the suppressive effect of PDPs on plasmid populations. To test our hypothesis, we challenged populations of *Escherichia coli* carrying the RP4 plasmid with the PRD1 phage under conditions where opportunities for conjugation were either weak or strong by manipulating the rate of influx of plasmid-free immigrant cells into bacterial populations, a manipulation first used in a seminal paper by Dimitriu and colleagues [35]. We quantified the population dynamics of bacteria, plasmids, and phage to understand the ecological consequences of this manipulation, and we then used extensive phenotyping and genome sequencing to study the evolutionary response to phage under variable immigration treatments.

## Materials and methods

### Bacterial, plasmid, and phage strains

To investigate the eco-evolutionary interaction between phage and plasmid, we used the plasmid-dependent phage (PDP) PRD1 and the promiscuous, multidrug-resistant (MDR) plasmid RP4. To enable high-resolution monitoring of plasmid dynamics during experimental evolution, RP4 was engineered with a GFP fluorescent marker. Briefly, the *gfp* cassette, incorporating its own promoter and terminator, was amplified from a synthetic template and integrated into *SnaBI* and *SbfI* linearised RP4 via a Seamless Cloning reaction (see Supplementary Table 4 for specific cloning parameters). The resulting RP4:GFP construct was initially transformed into *E. coli* DH5α by electroporation before being conjugatively transferred into *E. coli* MG1655, which served as the donor strain for all subsequent experiments. The plasmid-free *E. coli* strain J53 was employed as the recipient. J53 is a preferred choice for conjugation assays due to its high resistance to sodium azide, which facilitates efficient counterselection against donor strains without the need for further genetic engineering. As ubiquitous laboratory ‘workhorses’, these K-12 lineage strains provide a robust and well-characterized genetic background, making them ideal models for the short-term experimental evolution studies conducted here.

### Experimental evolution setup

**Figure 1:**
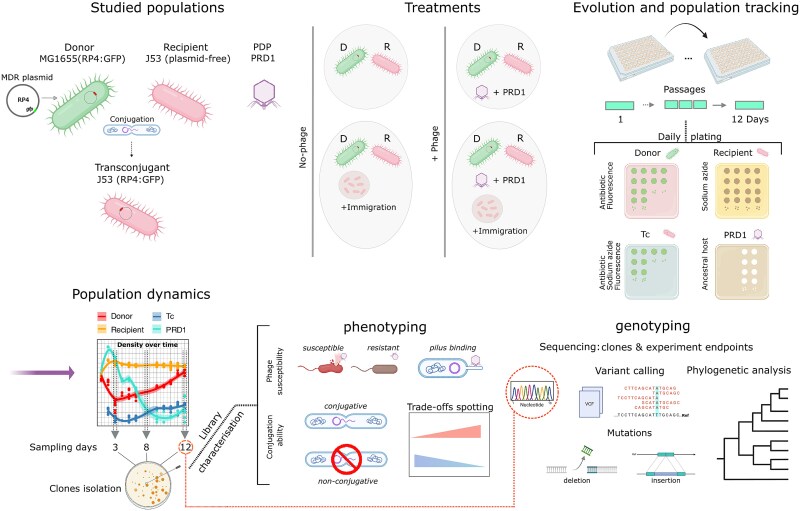
Experimental workflow for evaluating plasmid–phage eco-evolutionary dynamics. The interaction between the plasmid-dependent phage PRD1 and the multidrug-resistance plasmid RP4 (GFP-marked) was investigated by co-culturing *E. coli* MG1655 (plasmid donor) and J53 (plasmid-free recipient) strains under four conditions: control, PRD1 addition (single initial addition; MOI ≈ 10), recipient immigration (daily addition of ~10^7^ fresh J53 cells), and their combined effect. Populations were propagated via daily serial passage over 12 days, with donor, transconjugant (Tc), recipient, and phage densities monitored daily via selective plating. Evolutionary outcomes were assessed through genomic sequencing and mutational scanning of endpoint populations and individual clones isolated at Days 3, 8, and 12. Subsequent phenotyping of evolved clones for phage susceptibility, conjugation ability, and phage–pilus binding was performed to identify the mechanistic basis of evolutionary trade-offs (this figure was created with BioRender.Comand and Inkscape).

The evolutionary impact of PRD1 on RP4 was investigated by co-culturing *E. coli* MG1655 (plasmid donor) and J53 (plasmid-free recipient) under four conditions: control, PRD1 exposure, recipient immigration, and their combination. The immigration treatment consisted of a daily addition of ~10^7^ fresh plasmid-free J53 cells, intended to increase opportunities for plasmid conjugation in the system. Co-cultures were initially established by mixing independent overnight cultures of each strain (~1 × 10^7^ CFU/ml donors and ~2 × 10^7^ CFU/ml recipients; donor-to-recipient ratio of 1:2). PRD1 was added at ~1 × 10^8^ PFU/ml (single initial addition; MOI ≈ 10). Evolution experiments for all conditions were conducted simultaneously in triplicate in 96-well plates containing LB Lennox broth and incubated at 37°C under static conditions. Each daily passage culture had a final volume of 200 μl. Potential cross-contamination between treatments and replicates was minimized by leaving gaps between replicates and two full rows between treatments. These gaps were filled with sterile LB to monitor for any potential cross-contamination between samples. Populations were propagated by daily serial passage (1:10 dilution into fresh medium) for 12 days. In the control condition, phage and immigration were omitted. See Fig. 1 for the experimental design.

### Monitoring of plasmid and phage dynamics

Population dynamics were monitored daily over 12 days by quantifying colony-forming units (CFUs) for bacterial populations and plaque-forming units (PFUs) for PRD1. We employed a selective plating regime to differentiate subpopulations: donors were selected on LB agar supplemented with ampicillin (100 μg/ml), recipients on sodium azide (100 μg/ml), and transconjugants using a combination of both. As ampicillin selects for all plasmid-bearing cells (donors and transconjugants), donor density was estimated by subtracting the transconjugant count from the total plasmid-positive population. Briefly, culture aliquots were serially diluted (10^−1^ to 10^−8^) in PBS supplemented with 5 mM sodium citrate to minimize phage adsorption or in phage buffer for PRD1. Subpopulations were quantified using the drop-plate method (20 μl per dilution), whereas PFUs were determined by spotting dilutions onto bacterial lawns of the ancestral RP4 host. All assays were performed in duplicate and incubated at 37°C for 24 h. GFP fluorescence was used to verify plasmid maintenance in donor and transconjugant colonies. To distinguish whether phage decline was due to host resistance or low inoculum, a high dose of PRD1 (≈1 × 10^8^ PFU, MOI ≈ 10) was reintroduced on Day 7 in a subset of parallel samples, with subsequent dynamics monitored as described. Phage-mediated effects on host populations were analysed using the log₁₀ response ratio (LRR), a robust metric for quantifying effect sizes across multifactorial treatments (i.e. PRD1 phage + immigration). By calculating the LRR of daily bacterial doublings relative to no-phage controls, we isolated the suppressive effect of PRD1 across immigration treatments. To assess whether competition with plasmid-free cells contributed to phage-mediated plasmid suppression, we performed an additional 3-day experiment to quantify the relative effects of competition and phage killing. We tested the independent and combined effects of J53 recipient cells and PRD1 on both phage-susceptible MG1655(RP4:GFP) (conjugation-proficient) and phage-resistant MG1655(RP4:trbE mutant) (conjugation-deficient) hosts to isolate the impact of each factor. Strain densities were monitored daily by selective plating as described. Plasmid loss in colonies of the conjugation-proficient strain was detected by the simultaneous loss of plasmid-encoded GFP fluorescence and antibiotic resistance markers. The calculation of bacterial generations was based on the formula $n={\log}_2\left(\frac{N_{\mathrm{f}}}{N_{\mathrm{i}}}\right)$ where *n* is the number of generations, *N*_f_ is the final population size, and *N*_i_ is the initial population size. For the treatment without phage, we have

Day 1: ${n}_1={\log}_2\left(\frac{10^9}{10^7}\right)$, Days 2–12 (1:10 dilution): ${n}_{2-12}={\log}_2\left(\frac{10^9}{10^8}\right)\times 11$


$$ {n}_{1-12}\approx 43\ \mathrm{generations} $$

### Phenotypic characterization of evolved plasmid hosts

To assess the outcomes of the RP4–PRD1 interaction, we evaluated the frequency and degree of phage resistance in a library of 580 purified donor and transconjugant clones (360 from phage-treated populations and 200 from conditions without phage), sampled on Days 3, 8, and 12 (30 colonies per population per time point). Phage susceptibility was determined by plaque assays and classified into three categories: (i) susceptible—similar to the ancestral RP4 host; (ii) partial resistance—producing cloudy plaques and showing resistance levels 5–1000-fold higher than the ancestral host; and (iii) completely resistant—showing neither plaques nor lysis zones even under high phage densities (Fig. 3A).

A representative panel of 65 clones, encompassing all three identified phenotypes, was selected to evaluate the fitness costs associated with phage resistance. Conjugation ability of the evolved clones was assessed using standard filter mating assays on LB agar (24 h at 37°C) with a donor-to-recipient ratio of 1:2. To ensure consistent cell densities across assays, cultures were adjusted to a uniform OD600 before mating. Post-incubation, cells were recovered from filters, resuspended, and serially diluted (10^−1^ to 10^−8^) in PBS, and all dilutions were then spotted onto selective LB agar plates. Detection of transconjugants was based on both antibiotic selection and fluorescence markers. When the evolved clone was a derivative of MG1655(RP4:GFP) (original ancestral donor strain), J53 served as the recipient, and transconjugants were selected on LB agar containing ampicillin (100 μg/ml) and sodium azide (NaAz, 100 μg/ml). When the evolved clone tested as a donor was J53(RP4:GFP) (transconjugants generated during the experiment), MG1655 RFP-tagged chromosomally was used as the recipient. In the latter, transconjugants were identified via dual fluorescence: donors appeared green (GFP), whereas transconjugants appeared yellow due to the colocalization of GFP and RFP signals. The limit of detection for transconjugants was estimated to be between 10^−3^ and 10^−4^. Phage susceptibility was quantified using the efficiency of plaquing (EOP), calculated as the mean plaque titre of the evolved clone divided by the mean titre of the ancestral RP4 host. Conjugation efficiency was calculated using an analogous ratio based on transconjugant counts (Figs 3, 4 and 5). All assays were performed in three independent biological triplicates, each containing two technical replicates.

### Genotypic characterization of evolutionary outcomes

To investigate the evolutionary trajectories towards phage resistance, we sequenced and analysed both mixed endpoint populations from the evolution experiments and individual mutant clones displaying different phage susceptibility and conjugation phenotypes. Genomic DNA was extracted using the DNeasy Blood and Tissue Kit (QIAGEN), and plasmid DNA using the PureLink Quick Plasmid Miniprep Kit (Invitrogen), both following the manufacturer’s protocols. Sequencing was performed on a NovaSeq 6000 System with 2 × 150 bp paired-end reads (GENEWIZ UK Ltd). Illumina reads were quality filtered (Trimmomatic) and used to generate plasmid *de novo* assemblies for individual clones with SPAdes [40] v3.15.2.

Mutations were identified in both mixed populations and plasmid assemblies using Breseq [41] v0.37.1 (https://github.com/barricklab/breseq). The option to predict polymorphic mutations was used to analyse the mixed population, and default parameters were used for the individual clones, which were additionally scanned with Snippy v4.6.0 (https://github.com/tseemann/snippy)**.** In both cases, the ancestral RP4 sequence was used as the reference to determine mutation positions and their predicted effects on proteins. Insertion of IS421 was identified through manual inspection of annotated sequences generated with BAKTA [42] v1.11. Chromosomal mutations were assessed similarly, using the complete genomes of MG1655 (donors) or J53 (transconjugants) as references. Reference genomes were assembled using ONT long reads (PLASMIDSAURUS Ltd) and polished with Illumina reads (GENEWIZ from Azenta Life Sciences) pypolca v0.2.0 (https://github.com/gbouras13/pypolca). The protein structures of *virB4/trbE* mutant variants were predicted with ColabFold [43] v1.5.5 (https://github.com/sokrypton/ColabFold), combining AlphaFold2 and MMseqs2. Visualization and structure sequence alignment comparison analysis was performed using ChimeraX [44] (v1.9).

### Analysis of bacterium–phage interaction with electron microscopy

Transmission electron microscopy (TEM) was used to examine interactions between phage PRD1 and *E. coli* carrying RP4 derivative clones associated with either phage susceptibility or resistance. Clones with single mutations in *trbI* or *trbE* were selected as representative resistance genotypes, whereas a susceptible clone without plasmid mutations and plasmid-free *E. coli* J53 cells served as positive and negative controls, respectively.

Briefly, a single fresh colony grown overnight on LB Lennox agar plates was lifted onto a sterile loop and gently resuspended in an Eppendorf tube containing 100 μl double-distilled water, turning it very slightly turbid. Then, 5 μl of the cell suspension was added to the surface of a freshly glow-discharged carbon-coated Formvar EM grid and left for 30 s to allow settling of bacteria. The grid was gently blotted and 5 μl of neat PRD1 stock was then added and incubated for a further 30 s at room temperature. The grid was blotted again, negatively stained with 5 μl of 2% aqueous uranyl acetate, and immediately blotted once more. Grids were analysed on a 120-kV Tecnai Spirit Biotwin TEM at ×20 000 magnification. Images were recorded using a F4.16 Tietz CCD. For each RP4 derivative clone, interactions were assessed in two independent experiments. Phage binding was quantified by counting the number of viral particles attached per cell from 20 independent observations.

For scanning electron microscopy (SEM) of phage–pilus interaction, 10 single fresh bacterial colonies (grown overnight on an LB Lennox agar plate) were each covered with 5 μl of neat PRD1 phage stock (~1 × 10^11^), incubated for 30 s at room temperature, and gently rinsed three times with 0.1 M PBS. The colonies were then fixed on the plate with 1% paraformaldehyde and 2% glutaraldehyde in 0.1 M PBS for 1 h, rinsed three times in 0.1 M sodium cacodylate, and post-fixed by layering 1% cacodylate-buffered osmium tetroxide and 1% aqueous thiocarbohydrazide, rinsing thoroughly in between. Individual blocks of agar 5 mm × 5 mm, containing single colonies, were cut from the plate and dehydrated through a graded ethanol series (30%, 50%, 70%, 95%, and 3 × 100%, 20 min each) and then dried in a Leica EM CPD 300. The samples were then coated with 6 nm of evaporated gold in a Leica ACE 600 sputter coater. Individual colonies were analysed and imaged in a Hitachi SU8030 scanning EM.

### TrbE/VirB4 distribution and phylogenetic analysis

IncP-group plasmids were identified from a global collection of plasmid genomes that integrates multiple plasmid databases [45]. Metadata for these plasmids (e.g. mobility, host, AMR gene content) was also retrieved as part of the collection. We searched the database via BLASTp [46] using the protein sequence of TrbE/VirB4 from plasmid RP4 as a query. We restricted the matches to plasmids annotated as ‘IncP’ according to the metadata available for the database (*n* = 315). From these, we only kept plasmids with TrbE/VirB4 matches displaying at least 60% sequence similarity (*n* = 182) for downstream analysis.

A full-length TrbE/VirB4 protein alignment combining the 182 sequences identified from public archives and 10 representative variants from our dataset was generated with muscle v3.8.1551 [47] using the ‘-sv -maxiters 2’ options. The alignment was processed with trimal v1.4.1 [48] with the ‘-gt 0.9’ parameter and a maximum-likelihood phylogeny was generated from it with raxml:tree from raxml-ng v1.1.0 [49] under the LG + G4 model using the following parameters: ‘--all --model LG+G4 --seed 12345 --tree rand{10},pars{10} --bs-trees 100’. The tree was visualized in iTOL [50] incorporating protein sequence length to illustrate the detected truncated variants. To ensure that TrbE truncations were not the result of annotation artefacts, we implemented several control procedures, including sequence-similarity identification (BLASTp) independent of existing feature annotations, genomic neighbourhood alignments across diverse IncP plasmids, and a comparative size analysis of the conserved conjugation protein TrbB as a negative control (Supplementary Figs 8–9).

## Results

### Immigration enhances the suppressive effect of plasmid-dependent phage

To study the impact of PDP treatment, we established *E. coli* co-cultures consisting of a plasmid-carrying strain (MG1655) and a plasmid-free strain (J53), which served as the donor and recipient of the plasmid RP4, respectively. RP4 is a highly conjugative multidrug-resistance (MDR) plasmid that is generally considered naturally derepressed and therefore largely permissive to PDP infection; however, this state is expected to vary with physiological conditions [51]. To manipulate the opportunity for plasmid conjugative transfer, populations were propagated through daily serial passaging under different levels of immigration of J53 recipient cells (0% or 50%). Bacterial and phage dynamics were monitored every 24 h for 12 days, corresponding to ~43 bacterial generations in the absence of phage and 46–56 generations under phage treatment, after which evolutionary outcomes were determined (see Fig. 1 for the experimental design and workflow).

**Figure 2:**
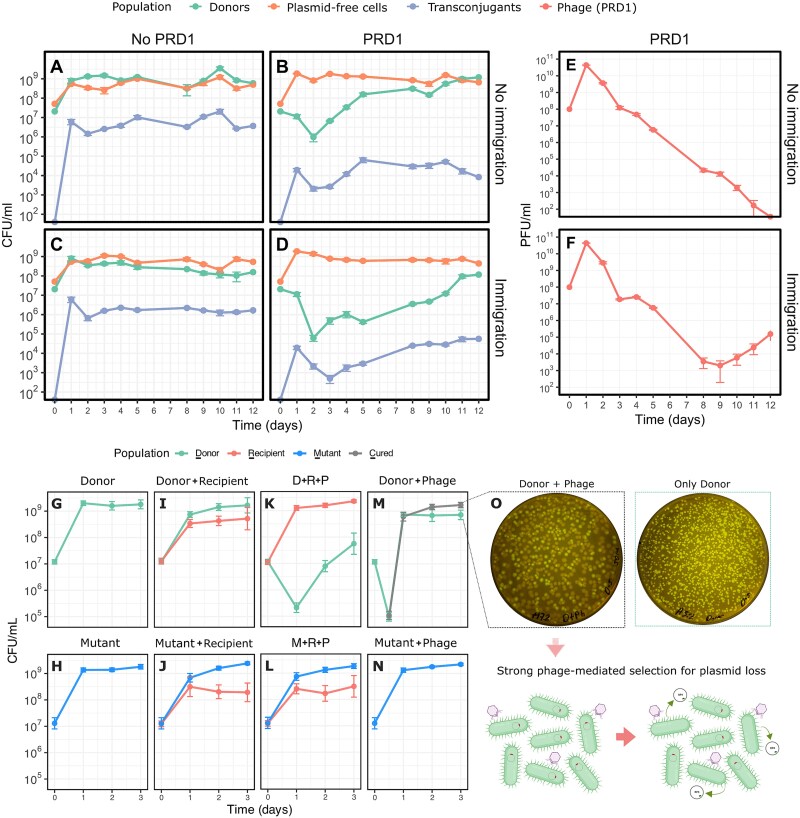
Immigration enhances phage-mediated plasmid suppression and prevents plasmid spread. Panels (**A**)–(**F**) show the mean density of plasmid donors, plasmid-free cells, transconjugants, and phage over time (± s.e.; *n* = 3) under the different conditions tested. (**A**) No-phage, no-immigration. (**B**) PRD1 treatment, no-immigration. (**C**) No-phage with immigration. (**D**) PRD1 treatment with immigration, whereas (**E**) and (**F**) correspond to the phage when immigration was absent or present, respectively. Treatment with phage suppressed the plasmid (main effect phage *P* = 1.94E−05), and this effect was enhanced by continual immigration of plasmid-free cells (phage × immigration interaction *P* = 2.32E−03) as assessed with repeated measures ANOVA. Immigration showed an impact on phage density only from Day 8 onwards (linear model with *post hoc* day × immigration interaction, *P* = 2.65E−04, Supplementary Tables 1, 2 and, 3). Panels (**G**)–(**O**) show the impact of competition and phage killing on donor growth dynamics (± s.e.; *n* = 3). Recipient competition alone attenuated donor growth by ∼2-fold by Day 1 (**I**–**J**) relative to monocultures (**G**–**H**). Whereas PRD1 exerted a potent but transient suppressive effect on donor populations (**M**), the addition of recipient cells significantly delayed recovery (**K**). The phage-resistant mutant remained unaffected by PRD1 alone (**N**) but experienced similar slight growth attenuation in the presence of recipients (**J**, **L**). Notably, PRD1 exposure imposed strong selection for plasmid loss, with ∼50% of the population losing both fluorescent and antibiotic resistance plasmid markers (**M**, **O**).

PRD1 treatment led to a rapid suppression of the RP4-bearing cells (donors and transconjugants), but this effect was transient, with plasmid densities eventually recovering to match phage-free controls. Immigration increased the short-term impact of phage treatment, delaying plasmid recovery until the end of the experiment (Fig. 2A–D, Supplementary Data 1), supporting the notion that the efficacy of PDP treatment is greatest when opportunities for conjugation are plentiful. However, phage densities were not significantly impacted by immigration (Fig. 2E and F), implying that the underlying strength of selection for phage resistance did not depend entirely on increased opportunities for conjugation.

### PRD1 suppresses plasmids by killing donor cells, selecting for plasmid loss and preventing plasmid transfer

To better understand the suppressive effects of PRD1 treatment, we assessed the dynamics of plasmid donors, recipients, and transconjugants (Fig. 2A–D, Supplementary Data 1). PRD1 exposure reduced donor and transconjugant populations (100–1000-fold reduction), an effect largely driven by the killing of the more prevalent donor cells (~100-fold higher abundance). This suppression remained transient without immigration but transitioned to a sustained state when fresh recipients were continuously introduced (Fig. 2B and D), highlighting a synergistic effect between PRD1 infection and immigration (Supplementary Fig. 2). PRD1 treatment also led to sustained suppression of transconjugants, independent of immigration rate, highlighting the ability of PRD1 to prevent the transfer of plasmids between bacterial strains. The stability of transconjugant densities in the no-phage treatments suggests that plasmid transfer occurred primarily early in the experiment and that the fitness cost of plasmid carriage in the transconjugant genetic background limited their population expansion.

We further investigated if competition with recipient cells facilitates phage-mediated plasmid suppression by testing the effects of PRD1 killing and recipient competition independently and in combination. Recipient cells alone moderately attenuated the growth of plasmid donors (∼2-fold; Fig. 2I and J) relative to monocultures (Fig. 2G and H), even when phage infection was precluded by the lack of a functional conjugation machinery in the resistant mutant strain (Fig. 2L). However, this competitive effect was transient, with donor cells typically outcompeting recipients after 24 h. Although PRD1 alone induced a sharp but temporary decline in donor density to ∼1%, followed by a near-complete recovery by Day 2 (Fig. 2M), the combination with recipient cells markedly delayed this recovery (Fig. 2K). This observation is consistent with the dynamics seen in our 12-day evolution experiments. In contrast, the phage-resistant mutant remained largely unaffected by phage treatment (Fig. 2N and L). Together, these results suggest that competition between plasmid-bearing and plasmid-free cells enhances the suppressive effect of PRD1.

PRD1 treatment also promoted large-scale selection for plasmid-free segregants in donor populations, with ~50% of the host population losing the RP4 plasmid. This was evinced by the simultaneous loss of plasmid-encoded GFP fluorescence and antibiotic resistance markers (Fig. 2M and O). These results highlight plasmid loss as a strong adaptive response to the high selective pressure imposed by PDPs.

### Immigration slows the evolution of phage resistance

**Figure 3:**
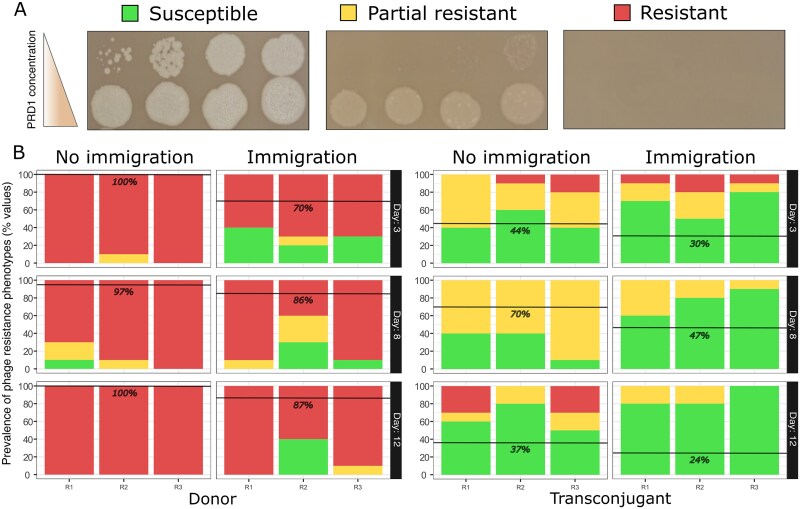
Prevalence of phage resistance in plasmid populations. (**A**) Representative results of PRD1 dilution spotting assays (10^−8^ to 10^−1^) on lawns of isolates from the evolution experiments, classified as resistant (dark red), partial resistant (yellow), or susceptible (bright green). (**B**) Distribution of these phenotypes among donor and transconjugant clones (bar plots on the left and right, respectively) from the three independent replicates of the evolution experiments (separate bars for R1–R3). Isolated clones from no-immigration and immigration conditions were collected on Days 3, 8, and 12 (indicated at the top and by the right-side annotations). Black lines show the percentage of resistant isolates for the three replicates at each time point. The proportion of phage-resistant isolates (shown at each panel as i.e. either full or partial resistance) was higher in the donor population in the absence of immigration at Day 3 (odds ratio = infinity, *P* = .0019), but not at Day 8 (odds ratio = 4.46, *P* = .353) or Day 12 (odds ratio = infinity, *P* = .112).

The rapid decline in phage abundance (Fig. 2E and F) and recovery of the plasmid-bearing donor strain (Fig. 2B and D) suggest that resistance to PRD1 evolved. To explore this further, we isolated 360 clones from phage-evolving populations and 220 clones from phage-untreated samples and tested their susceptibility to the ancestral PRD1 phage. Three distinct phenotypes were detected, including phage susceptibility (clear PRD1 plaques), partial PRD1 resistance (cloudy plaques), and complete resistance (no plaques) (Fig. 3A).

The donor strain rapidly evolved resistance to PRD1 in the absence of immigration, with >95% fully resistant isolates by Day 3, and this proportion remained stable across the experiment. In contrast, immigration delayed the evolution of phage resistance in the donor population; the proportion of resistant isolates was lower at Day 3 than at the end of the experiment (Fig. 3B, Supplementary Fig. 2). This pattern is consistent with our hypothesis that increased opportunities for conjugation reduce the strength of selection for PDP resistance, an effect that we identify is also driven by competitive interactions with plasmid-free recipients.

Phage suppressed minority transconjugant subpopulations throughout the experiment, suggesting that sensitivity to phage remained high in transconjugants compared to donors. Consistent with this idea, the frequency of resistance remained low in transconjugants across the entire experiment (Fig. 3B). Interestingly, phage resistance in transconjugants was usually partial, whereas the complete resistance phenotype was associated with donors.

In summary, isolate phenotyping revealed that resistance was high in donors compared to transconjugants and more prevalent in treatments without immigration. To further test these associations, we carried out a side experiment where we reintroduced a high density of PRD1 to populations at Day 7. Consistent with our phenotyping data, the suppressive effects of re-introducing PRD1 were most evident in the immigration treatment and on the transconjugants (Supplementary Fig. 1).

### Mutations in the conjugative pilus drive phage resistance evolution

**Figure 4:**
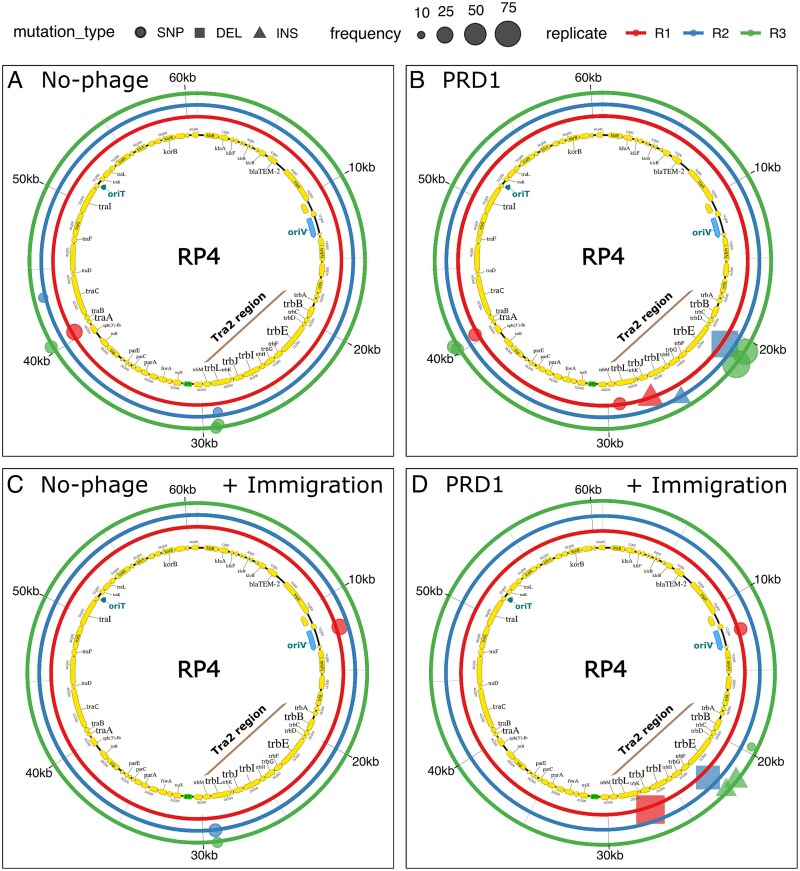
Plasmid population evolutionary responses to phage PRD1. Panels (**A**)–(**D**) show the genomic distribution and frequency of RP4 mutations detected at the endpoints of the evolution experiments across the tested treatments. The inner ring depicts the RP4 genomic map (with the Tra2 region highlighted, where most mutations clustered). The three outer rings represent biological replicates for each condition. Mutation types are indicated by symbols with shapes (circles = SNPs, squares = DELETIONS, triangles = INSERTIONS), with symbol size proportional to mutation frequency.

To investigate evolutionary responses to PRD1, we sequenced endpoint populations and tested for the evolution of the RP4 plasmid by quantifying the abundance of novel mutations present at a frequency of >10% in the plasmid population. By this stage, plasmid populations were largely dominated by the donor strain, which displayed high levels of PRD1 resistance (Figs 2 and 3). PRD1 treatment was associated with parallel evolution across populations in the Tra2 region, which encodes the structural components of the type IV secretion system (T4SS), also known as the conjugative pilus. Most high-frequency mutations were SNPs or indels in *trbI*, *trbJ*, and *trbE*, reaching population frequencies of 30%–77% (Fig. 4B and D). By contrast, in treatments without PRD1, no mutations in Tra2 exceeded 20% frequency, indicating that selection for altered conjugative ability is weak in the absence of phage (Fig. 3A and C). Low-frequency polymorphisms (10%–20%) were also detected in other plasmid regions, including *traA* and *oriV*, without a clear association with phage exposure or immigration treatments.

### Evolutionary trajectories to phage resistance and associated trade-offs

**Figure 5:**
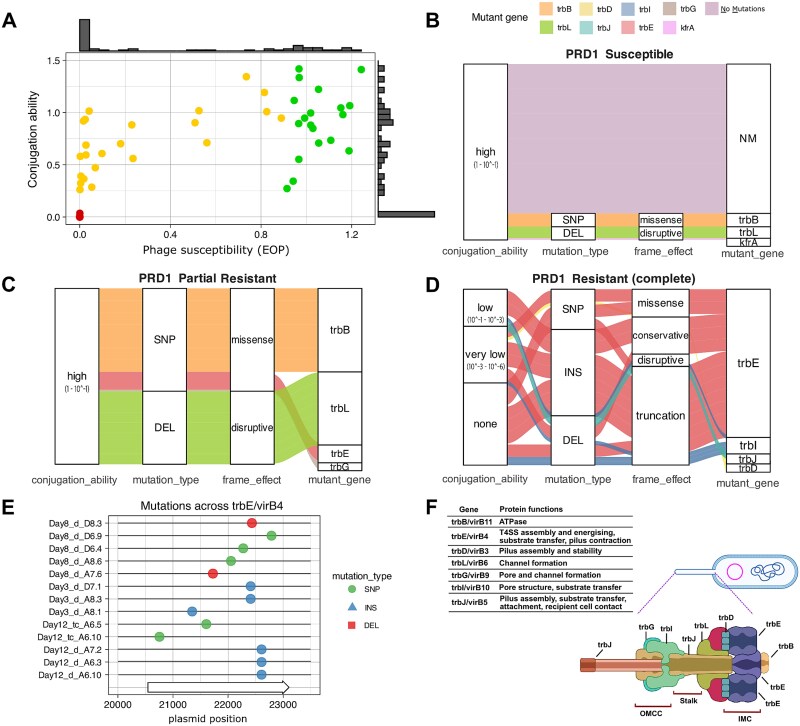
Phage resistance phenotypes and associated genotypes. (**A**) Scatter plot of phage susceptibility (EOP, *x*-axis) versus conjugation ability (*y*-axis) for 65 RP4-carrying *E. coli* clones from the evolution experiments. Dots represent mean phage susceptibility values across three replicates, ranging from resistant to partial resistant and susceptible. Values are relative to the ancestral RP4. Bar plots along each axis show the frequency distribution of phenotypes. Panels (**B**)–(**D**) illustrate the relationships between phage susceptibility, conjugation phenotypes, and plasmid mutations, including the affected genes, mutation types (SNP, DELETION, INSERTION), their impact on the reading frame, and cases where no mutations were detected (NM). Connecting lines indicate the mutated genes associated with each phenotype. Phenotype and genotype associated with (**B**) PRD1-susceptible clones. (**C**) Partial resistant clones. (**D**) Fully resistant clones. (**E**) Distribution of mutations targeting *trbE/virB4*, the most frequently mutated gene among individual clones. (**F**) Schematic of the type IV secretion system (T4SS) with regions showing the positions and predicted functions of proteins encoded by genes found to be mutated under PDP pressure.

To better understand the evolutionary responses of RP4 to PRD1, we further characterized 65 clones that were chosen to represent the full range of observed phage susceptibility phenotypes. To test for a trade-off between PRD1 resistance and conjugative ability, we quantitatively measured the phage susceptibility and conjugative ability of this panel of clones. As expected, we found a trade-off between phage resistance, measured as efficiency of plaquing, and conjugative ability, measured as relative transconjugant density in phage-free mating experiments. However, it is clear from this assay that phage resistance and conjugative ability are not binary traits, highlighting the ability of selection to fine-tune these traits (Fig. 5A, Supplementary Figs 3, 4 and 5, Supplementary Data 1).

To investigate the molecular basis of this trade-off, we sequenced 54 evolved plasmid-carrying clones. As expected, phage-susceptible clones, which were recovered from no-phage and the phage treatments, were not associated with plasmid mutations (Supplementary Data 2). Yet, some susceptible clones from the phage treatment were the exceptions, showing mutations in the genes *kfrA*, *trbB*, or *trbL*, but these mutations did not significantly affect conjugation (Fig. 5B). Clones with a partial phage resistance phenotype were mainly associated with mutations in *trbB*, an ATPase that powers pilus retraction and extension, or *trbL*, which is involved in channel formation for the translocation of DNA. In all cases, these mutations were associated with a moderate decrease in conjugative ability, highlighting the capacity of plasmid RP4 to evolve increased resistance to PRD1 without completely sacrificing conjugative ability (Fig. 5C).

Complete resistance to PRD1 was associated with mutations in *trbI*, *trbJ*, and *trbE*, matching the mutations identified by population sequencing (Fig. 5D), and consistent with previous reports identifying *trbE* as a major target for PRD1 resistance [27]. We identified a wide diversity of mutations in *trbE*, including SNPs, INDELS, and insertions caused by transposition of the IS421 element from the MG1655 chromosome into the plasmid, underscoring the role of IS elements in inactivating conjugative genes and driving rapid plasmid evolution [52]. Despite differences in mutation type and position (Fig. 5E), most were predicted to produce a truncated and misfolded TrbE protein (Supplementary Fig. 6).

TrbE plays key roles in the assembly and function of the T4SS, including inner membrane core complex (IMCC) assembly, energy supply, pilus contraction, and substrate translocation [53] (Fig. 5F). Consistent with this role, *trbE* mutations were associated with impaired conjugative ability. However, their effects were variable, and ~50% of clones carrying *trbE* mutations retained some level of conjugative ability, highlighting the complexity of the trade-off between conjugative transfer and PRD1 resistance.

We also scanned these clones for chromosomal mutations to assess whether chromosomally encoded genes might contribute to the observed phenotypic outcomes. This analysis revealed that only 20% of the resistant clones had acquired chromosomal mutations. The affected genes were functionally diverse and scattered across different regions of the genome, and we could not identify any associations between these mutations and the phenotypes of interest (Supplementary Data 2).

A small subset of evolved clones (7/54) exhibited a marginal increase in conjugation frequency (0.2–0.4 log-fold) or phage susceptibility (6/54, ∼0.2 log-fold) relative to the ancestral strain (Fig. 5A, Supplementary Fig. 5). As these phenotypic shifts were not associated with detectable plasmid mutations, they likely reflect measurement error or host chromosomal or epigenetic changes rather than plasmid evolution.

### Pilus is essential for phage-host infection

To investigate how the plasmid-encoded mutations associated with phage resistance affect the interaction with the bacterial host, we examined by TEM *E. coli* cells carrying RP4 variants associated with either susceptibility or resistance to PRD1 and quantified phage–host binding. Representative resistant clones carried mutations in either *trbI* or *trbE*, whereas a susceptible clone lacking plasmid mutations served as a control. The plasmid-free strain J53 was included to evaluate the host’s background phage interaction level. In all cases, the plasmid-carrying isolates used in this assay lacked any chromosomal mutations.

**Figure 6:**
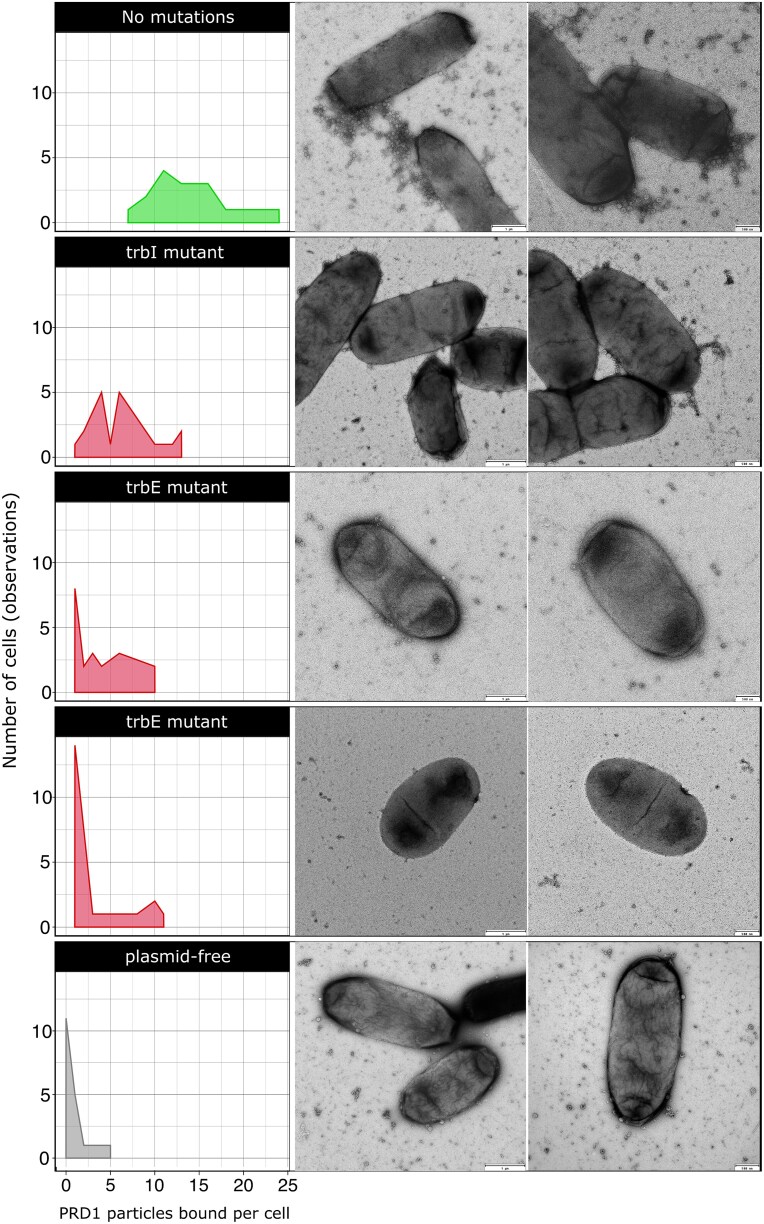
Bacterium–phage interactions via the conjugative pilus. TEM images show binding of phage PRD1 to *E. coli* cells carrying RP4 variants that are either phage-susceptible (top) or resistant (middle), with plasmid-free cells included as controls (bottom). Binding levels correlated with pilus presence: Susceptible cells displayed abundant phage attachment, whereas resistant mutants with impaired pilus formation bound fewer viral particles. Frequency distribution plots at the left quantify PRD1 binding (*x*-axis) across cells (*y*-axis). The mutated gene of each variant is shown above the corresponding plot. Images on the left include a 100-nm scale bar, whereas those on the right include a 50-nm scale bar to provide higher magnification of the interaction phenotype.

TEM examination of 20 individual cells per clone (analysed in two rounds of independent experiments) revealed that PRD1 binds abundantly to the conjugative pilus—mostly to the sides of the pilus (Fig. 6; clone without mutations at the top, Supplementary Fig. 7), a detail that remained unclear in earlier studies [54]. The susceptible clone displayed high phage binding, averaging 14 viral particles per cell, compared to resistant mutants, which bound far fewer phage—around 6 particles per cell for the *trbI* mutant and 2–3 for the *trbE* mutants (Fig. 6). In line with the above, the *trbI* clone retained pilus remnants, whereas *trbE* mutants lacked these, likely explaining the higher binding in the former. Phage attachment to plasmid-free J53 cells was minimal (15× lower than the susceptible clone), consistent with weak, nonspecific membrane interactions.

We also observed that cells from the susceptible clone began to lyse as early as 10 min after exposure to the phage, which contrasted to the resistant mutants that remained intact throughout the examination. This rapid onset of cell bursting in the susceptible strain reinforces the phenotypic differences and highlights the protective effect conferred by the phage resistance-associated mutations.

### Truncations in the pilus assembly protein occur in natural IncP plasmid populations

**Figure 7:**
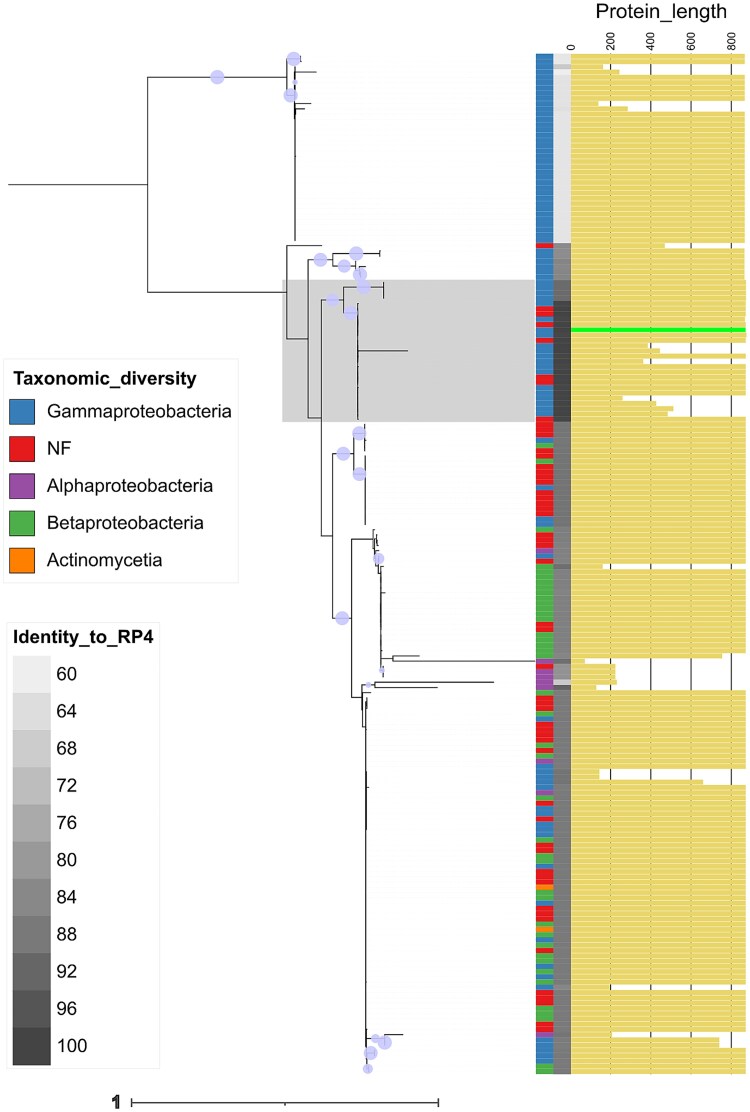
TrbE/VirB4 diversity in IncP plasmids. Phylogenetic tree of 194 TrbE/VirB4 homologs, including RP4 (length bar in bright green) and its evolved mutants from our evolution experiment—the region where they are distributed is highlighted. Protein length is shown by yellow bars (0–852 aa) on the right; shorter variants are present across multiple branches. Host taxonomic class is indicated by a colour-coded bar (NF indicate not found), followed by sequence identity to RP4 (60%–100%, grey gradient). Bootstrap support is marked by purple circles, where size is proportional to value (80–100). Tree scale indicates the number of substitutions per site under the model (LG + G4).

Our experiments revealed that RP4 usually evolves resistance to PRD1 through mutations that truncate the protein TrbE, resulting in high levels of phage resistance and low conjugative ability. To determine whether similar genetic changes could be seen more broadly across publicly available IncP plasmid sequences, we investigated the diversity of TrbE/VirB4. A full-length alignment of 194 TrbE/VirB4 homologs—including the wild-type RP4 protein, representative mutant derivatives from our evolution experiment, and 182 homologs (≥60% sequence identity) retrieved from plasmid public archives—showed that protein length is generally well conserved, but also revealed notable variation, a broad taxonomic distribution encompassing multiple classes (Fig. 7), and strong association with AMR, including multidrug-resistance gene carriage (Supplementary Figs 10–11). Consistently, we identified numerous truncated variants scattered across diverse branches of the TrbE/VirB4 phylogenetic tree—24 out of 182 (~13%), excluding those from our evolution experiment, ranging from 67 to 846 aa. These truncated variants, lacking N-terminal or/and C-terminal sequences compared to RP4 TrbE/VirB4, were identified in diverse host genera, such as *Escherichia*, *Morganella*, and *Pseudomonas*, and are present in a large proportion of plasmids (54%) encoding AMR genes, including those conferring resistance to broad-spectrum beta-lactams or colistin.

## Discussion

Plasmid-dependent phages (PDPs) are abundant and diverse [28], but their impact on the evolution and ecology of plasmids remains poorly understood. In line with previous studies, we find that plasmids can evolve resistance to PRD1 through mutations that disable or impair the conjugative apparatus [27, 32]. Although the loss of conjugative ability provides a simple solution to circumvent PDPs, mathematical models predict that conjugation plays a key role in plasmid maintenance [4, 5, 55], and the loss of conjugation is associated with an elevated risk of plasmid lineage extinction [34]. Our work shows that PRD1 resistance evolves slowly under ecological conditions that create a benefit for conjugation (Fig. 2) because trade-offs between resistance and conjugative ability weaken selection for resistance (Fig. 5). This, in turn, increases the suppressive effect of the PRD1 phage on the RP4 plasmid population (Fig. 2D).

In addition to their suppressive effects on strains carrying plasmids, PDPs have the potential to block the horizontal transfer of plasmids between strains. As expected, PRD1 suppressed the transfer of the RP4 plasmid between strains of *E. coli*. Interestingly, levels of resistance to PRD1 in transconjugants remained low, despite the strong suppressive effect of phage on transconjugants. Because only conjugative plasmids can transfer, transconjugants must be biased towards conjugative plasmids compared to the donor population, and this inherent bias must help to explain the low levels of resistance in transconjugants. PRD1 pressure maintained transconjugants at a low population density (<10^4^ cells), leading to strong bottlenecks in the population during passages to fresh medium (~20–2000 cells transferred per passage). We speculate that the low effective population size prevented the evolution of PRD1 resistance in transconjugant subpopulations by limiting the emergence of mutant plasmids and increasing the stochastic loss of rare PRD1-resistant transconjugant lineages.

Even though a large number of genes are necessary for the formation of the RP4 conjugative pilus [27], we only observed evolution in a subset of genes (*trbE*, *trbI*, *trbJ*, *trbD*, *trbB*, *trbL*, *trbG*) that have well-described roles in pilus formation, DNA translocation, and pilus retraction (Fig. 5). Consistent with this, some phage-resistant mutants produced pili that provided a substrate for PRD1 attachment (Fig. 6), suggesting that pilus retraction and substrate transfer are required for PRD1 to successfully infect cells. Mutations that truncate TrbE were the dominant mechanism of RP4 adaptation to PRD1 in our experiments. The assembly of the conjugative pilus is associated with substantial fitness costs [37–39], and we speculate that TrbE truncation is a common evolutionary path to PRD1 resistance because the loss of TrbE prevents pilus formation, providing low-cost PRD1 resistance. The *trbE* gene also presents a large mutational target compared to the other genes in the Tra region (2559 bp), which may also help to explain why this evolutionary trajectory is common. We do not entirely exclude the possibility that chromosomal genes may play a secondary role in modulating phage susceptibility or conjugative ability; however, our results indicate that their contribution was minor under the conditions of our experiment. Co-culturing bacteria and phage typically results in rapid co-evolution mediated by changes in bacterial cell surface components and phage tail fibre proteins [56, 57]. PRD1 declined to very low phage densities (10^3^–10^4^ virions), making it difficult to test for phage evolution in endpoint samples using either genomic or phenotypic assays. Although phage adaptation in our system remains possible—particularly under conditions of recipient cells immigration that may favour phage adaptation to partially resistant bacterial hosts—the overall decline in phage density over time suggests that PRD1 was unable to co-evolve to infect phage-resistant bacteria. This implies that the likelihood of a canonical coevolutionary arms race in this system is low, as the primary resistance mechanism involves the plasmid loss or eliminating the functional conjugative pilus, which effectively removes the phage’s entry point.

Truncated variants of TrbE are common in IncP plasmids collected from diverse bacterial hosts and sources, including plasmids associated with antibiotic resistance genes (Fig. 7, Supplementary Figs 8–11). Notwithstanding the possibility that TrbE truncation reflects selection against the metabolic costs of pilus production, the observed patterns support a scenario where selection for resistance to PDPs may be widespread in nature. TrbE truncations in our study are associated with a dramatic loss of conjugative ability, suggesting that PRD1 may have played an important role naturally in preventing the dissemination of antibiotic resistance genes associated with IncP plasmids. One interesting avenue for research would be testing the hypothesis that the loss of conjugative ability in IncP plasmids is an evolutionary dead-end, as is suggested by large analyses of plasmids [34].

The anthropogenic use of antibiotics over the past century has driven the evolution of conjugative plasmids carrying AMR genes [45]. The acquisition of these plasmids in pathogens is associated with stemming from the metabolic burden of plasmid gene expression and disruptions to core cellular processes [1, 3]. Both theoretical and experimental studies show that conjugative transfer enables plasmids to persist in bacterial populations despite these costs, even under weak antibiotic pressure [5, 6, 8, 9]. Our results indicate that PDPs impose a strong selective pressure that limits the conjugative ability of plasmids and constrains horizontal transfer of resistance genes within microbial communities. The growing discovery of PDPs highlights their potential as a biological toolkit to suppress diverse conjugative plasmids [58]. From this perspective, our findings suggest that targeting microbial communities that serve as hotspots for plasmid transfer using PDPs could simultaneously eliminate antibiotic-resistant pathogens and push plasmids to evolve into less mobile forms, limiting the onward dissemination of resistance.

## Supplementary Material

Supplementary_material_wrag113

## Data Availability

Sequencing data generated during this study have been deposited in the NCBI Sequence Read Archive (SRA) and are available under *BioProject* accession: PRJNA1333424. *BioSample* accessions for mixed populations and individual clones are listed in Supplementary Data 2. Raw and processed data from this study are available in the Oxford University Research Archive (ORA) under DOI: 10.5287/ora-yrp6kzmjg
