# Supplementary material for: Eco-evolutionary responses to plasmid-dependent phage constrain the spread of multidrug-resistance plasmids
Source: ISME J. 2026 May 8;20(1):wrag113. doi: 10.1093/ismejo/wrag113 (PMC13222520; doi:10.1093/ismejo/wrag113)
Supplement: Supplementary_material_wrag113 [file supplementary_material_wrag113.zip › Supplementary_Figures&Tables.docx]

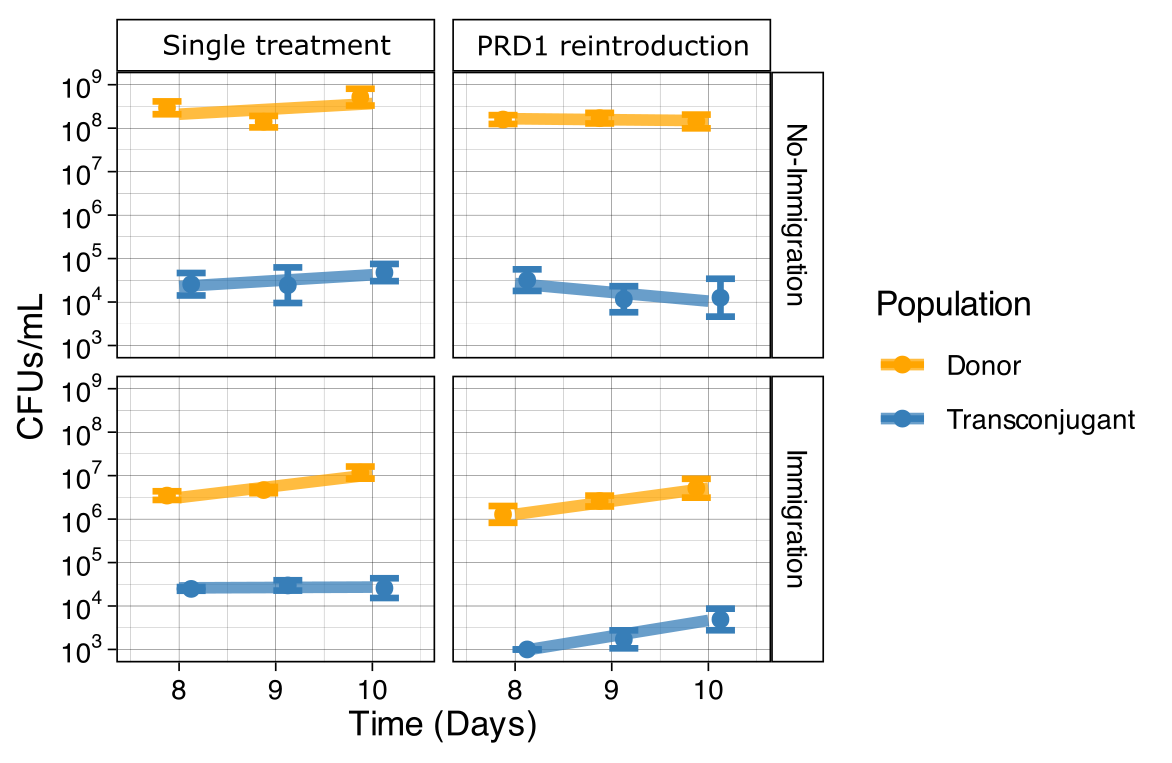


**Supplementary Figure 1. Effect of PRD1 reintroduction on plasmid host populations.** Phage reintroduction in parallel samples from the evolution experiments (right panels) caused a greater reduction in transconjugants (blue) than in donor cells (yellow), supporting a higher prevalence of phage resistance among donors. The effect was stronger under the immigration condition, consistent with reduced selection for phage resistance in this treatment.


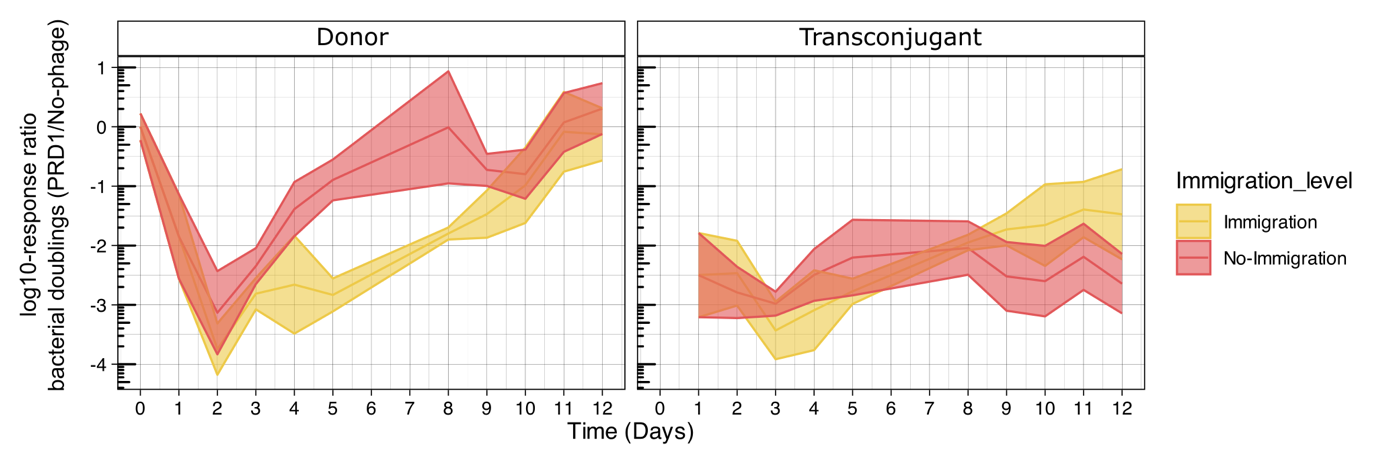


**Supplementary Figure 2. Bacterial doublings of plasmid hosts under PRD1 and immigration treatments.** The ribbon plots show the log10-response ratio of daily doublings by the donor strain (left panel) and transconjugants (right panel) in PRD1-treated cultures relative to untreated controls. Lines show the estimated response ratio, and the borders of the ribbon show the 95% confidence intervals in the response ratio.


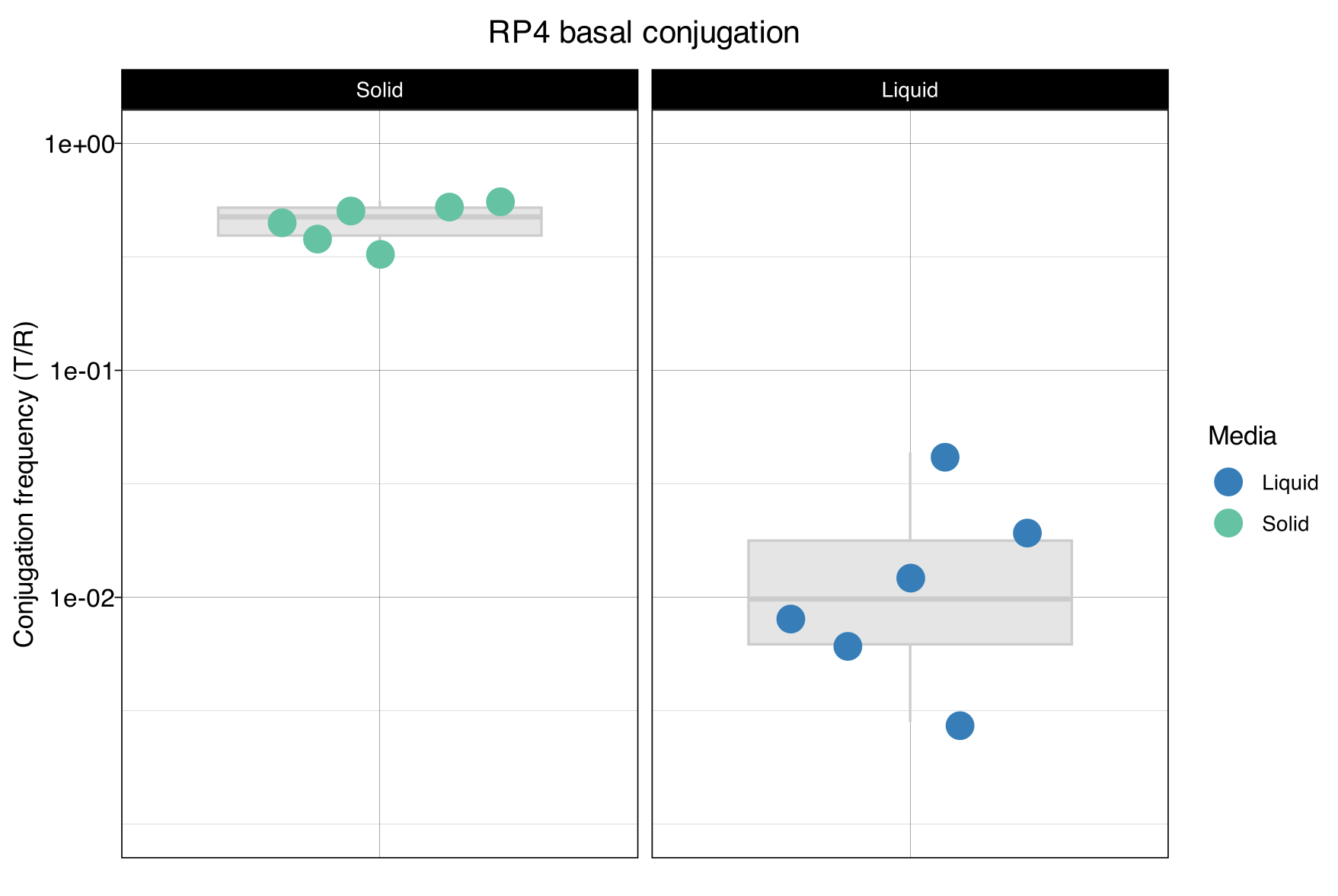


**Supplementary Figure 3. Conjugation efficiency of plasmid RP4.** Conjugation efficiency of RP4 was measured under solid and liquid culture conditions and calculated as the ratio of final transconjugants to recipients (T/R frequency). Each data point represents the average conjugation frequency from six independent replicates, and the box plots summarise the distribution of values. The basal conjugation frequency of RP4 was higher under solid culture conditions than in liquid culture, with values of approximately 0.5 and 0.01, respectively.


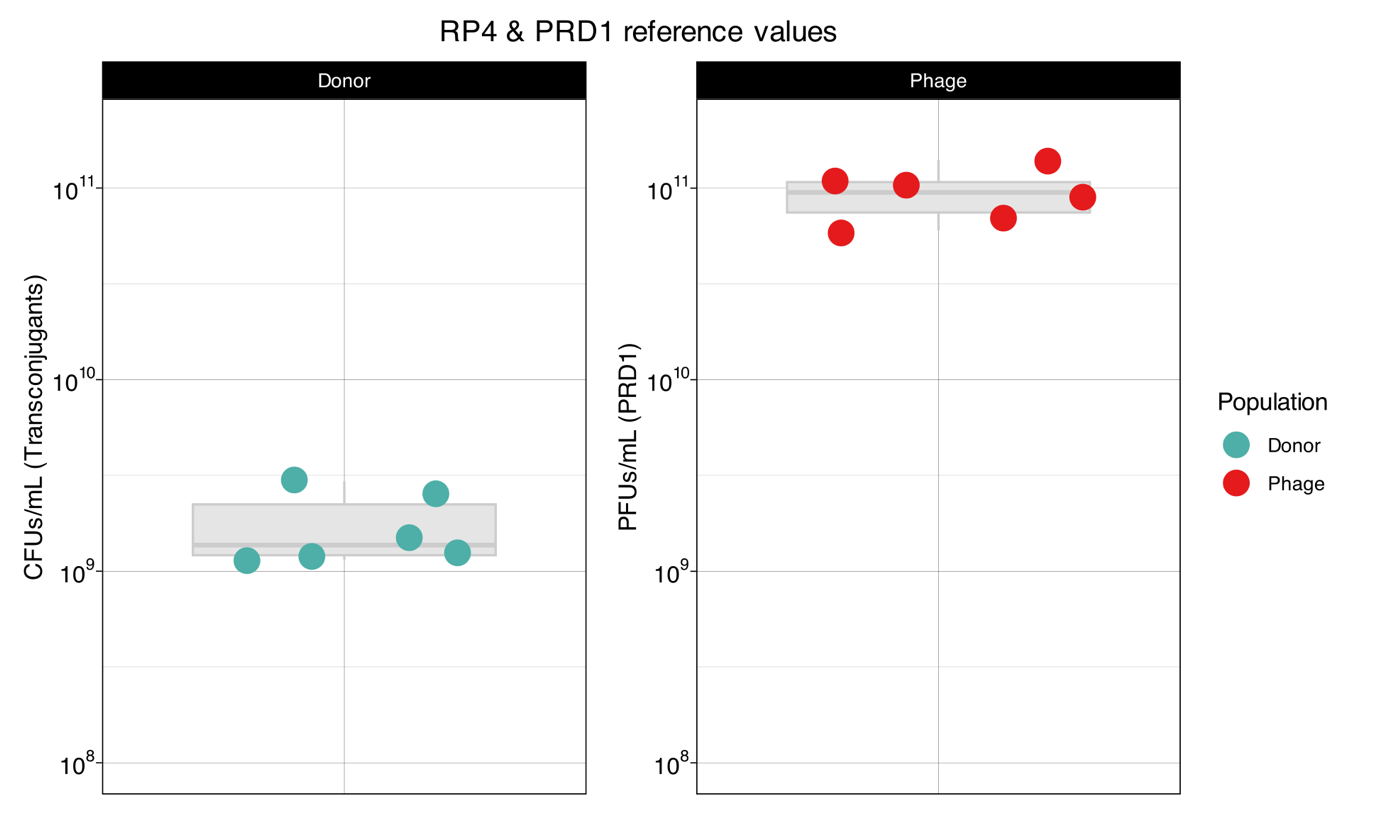


**Supplementary Figure 4. Reference values of ancestral RP4 used to estimate conjugation ability and PRD1 susceptibility.** The left plot shows the number of transconjugants (CFU/mL) obtained from filter matings using the *E. coli* strains MG1655 and J53 as the RP4 donor and recipient, respectively. The right plot shows the number of PRD1 plaques (PFU/mL) formed on a bacterial lawn of MG1655 carrying RP4 using a high-titer phage stock. Each data point represents the average value from six independent replicates.

**
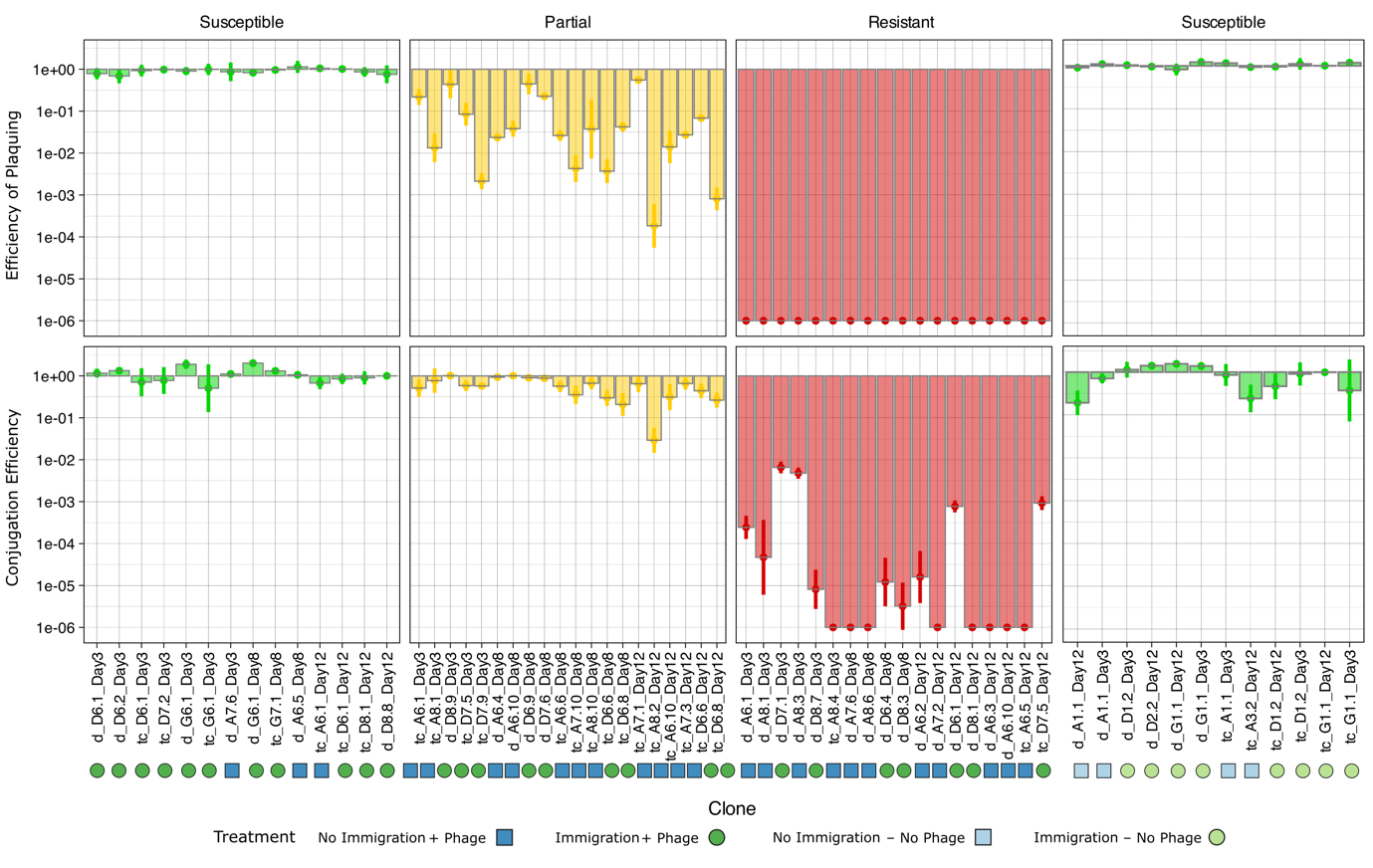
Supplementary Figure 5. Trade-off between phage resistance and conjugation ability.** Association between phage susceptibility (categories at the top) by efficiencies of plaquing and conjugation efficiency. Efficiency values (y-axis) are relative to the ancestral RP4 host. The individual clones tested (x-axis) are aligned to compare their outcomes in both phenotypes; the day from which they were retrieved is indicated on their labels.


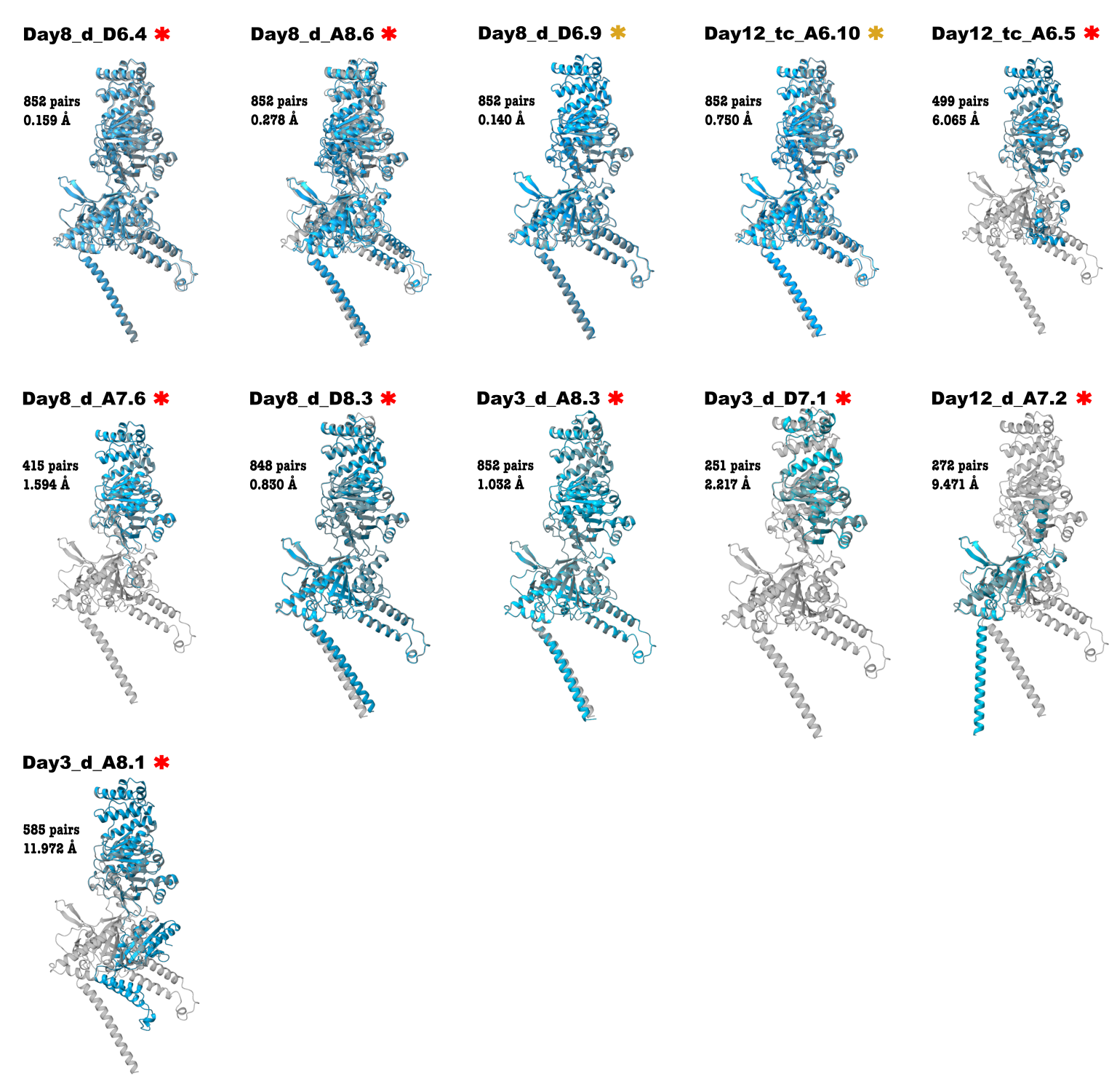


**Supplementary Figure 6. TrbE structure models from phage resistance mutant clones.** Mutant protein models (in blue) are shown on top of the wild-type version (in grey) for comparison. The number of atoms compared and the distance between the models (angstroms) are shown for each model. A red or yellow asterisk in the clone’s label indicates its association with either complete resistance or partial, respectively.


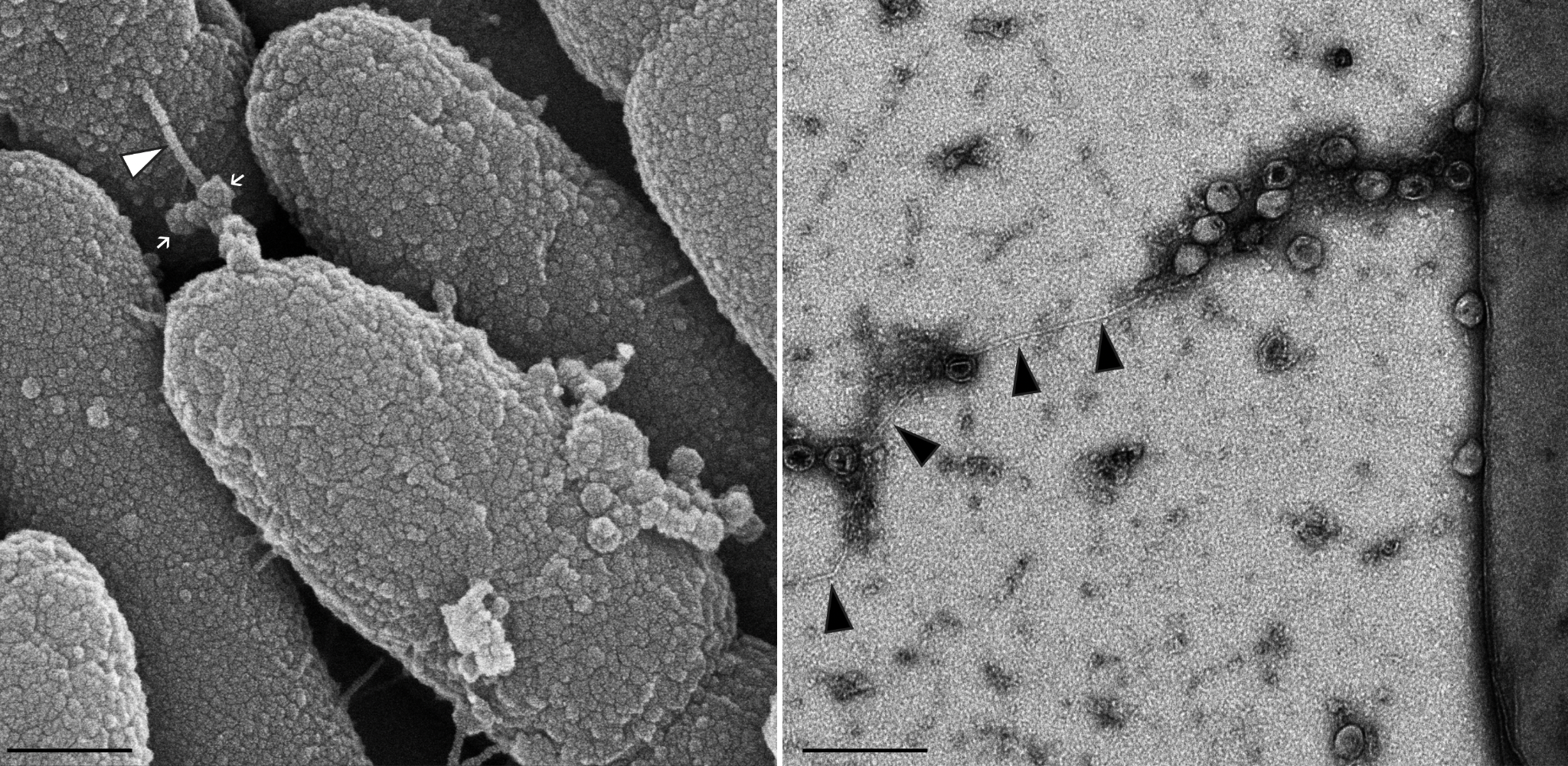


**Supplementary Figure 7. PRD1-pilus interaction.** Images on the left and on the right were generated by scanning electron microscopy (SEM) and transmission electron microscopy (TEM), respectively. White and black large arrows point to the pilus, small white arrows to the phage. Scale bar is 200 nm.


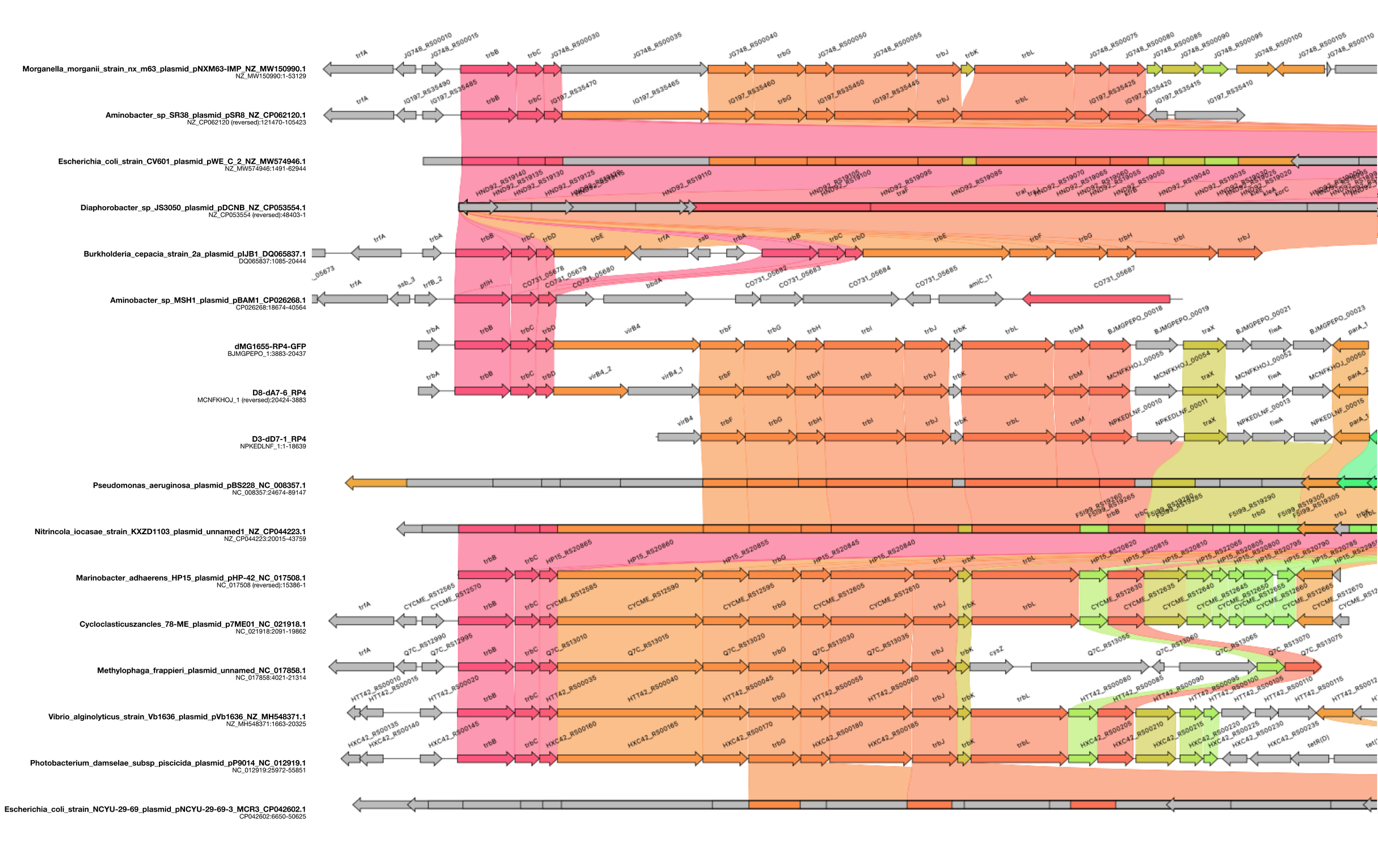


**Supplementary Figure 8. Protein alignment of RP4 and related IncP plasmids in the tra2 region.** The alignment shows the genomic neighbourhood surrounding TrbE across plasmids carrying either truncated or full-length versions of the protein. The visualisation confirms conserved synteny across plasmids and reveals potential signals of insertions, frameshift mutations, or rearrangements affecting TrbE and its genomic context.


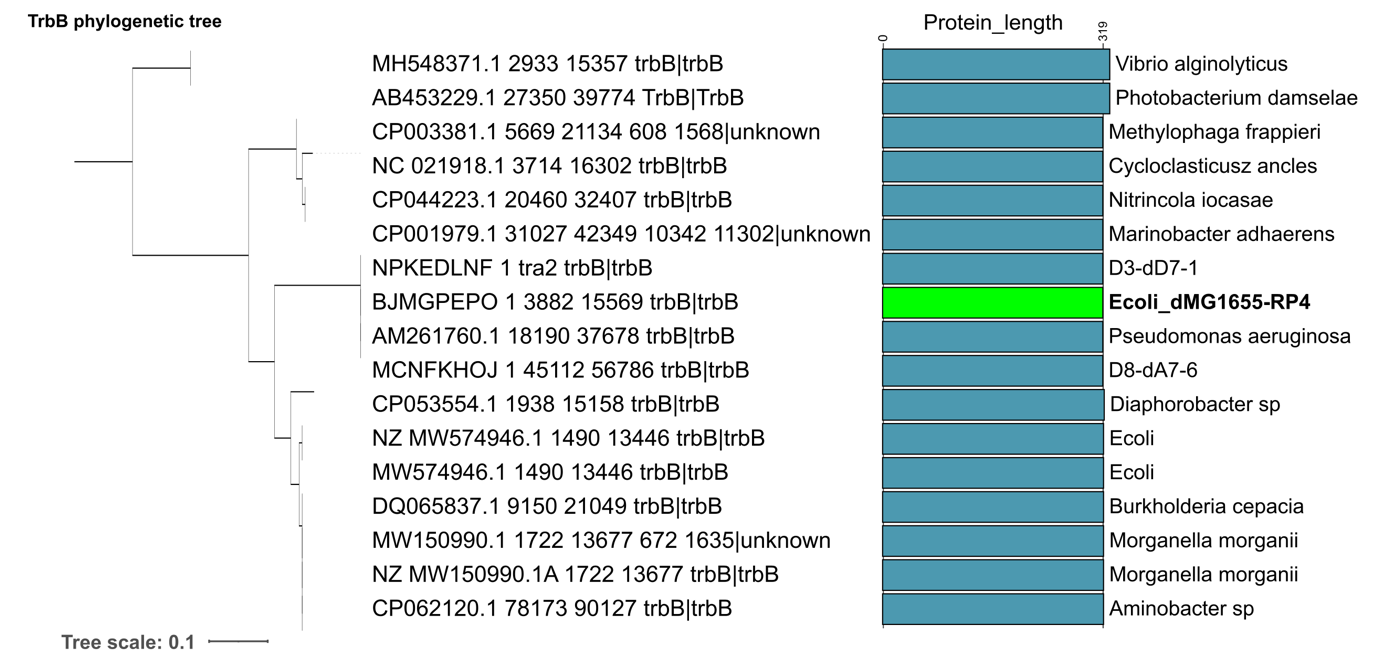


**Supplementary Figure 9. Phylogenetic and size analyses of the TrbB protein in RP4 and related IncP plasmids.** Alignment of the TrbB protein across RP4-related plasmids carrying TrbE truncations shows no detectable length variation, indicating that the observed truncations are specific to TrbE (see Figure 7).


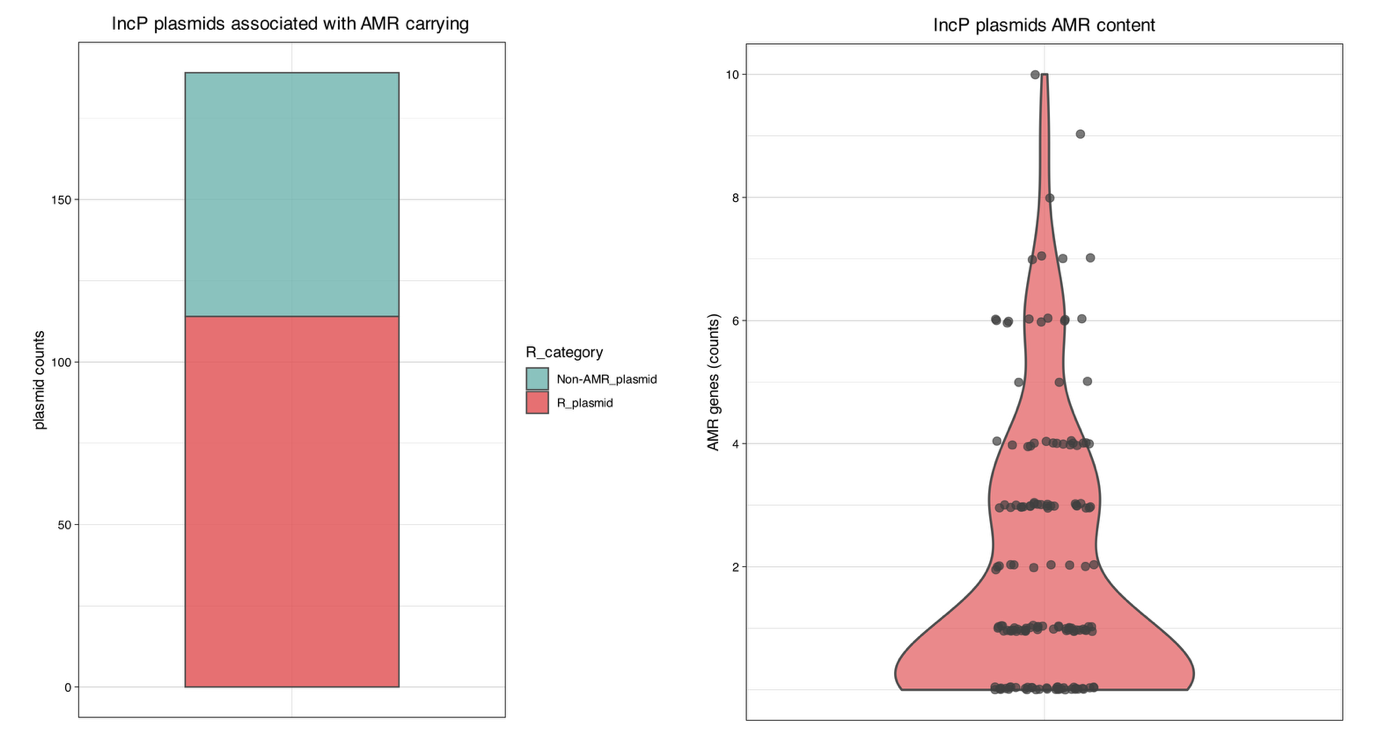


**Supplementary Figure 10. Association of IncP plasmids with AMR.**

RP4 close relatives (≥60% similarity) are strongly associated with AMR gene carriage (left panel), with 60% (115/194) carrying at least one AMR gene. The AMR gene content ranges from 0 to 10 genes, and a large proportion of AMR-positive plasmids (right panel; each dot representing an individual plasmid) carry more than two genes (62%), indicating that many are multidrug resistance plasmids.


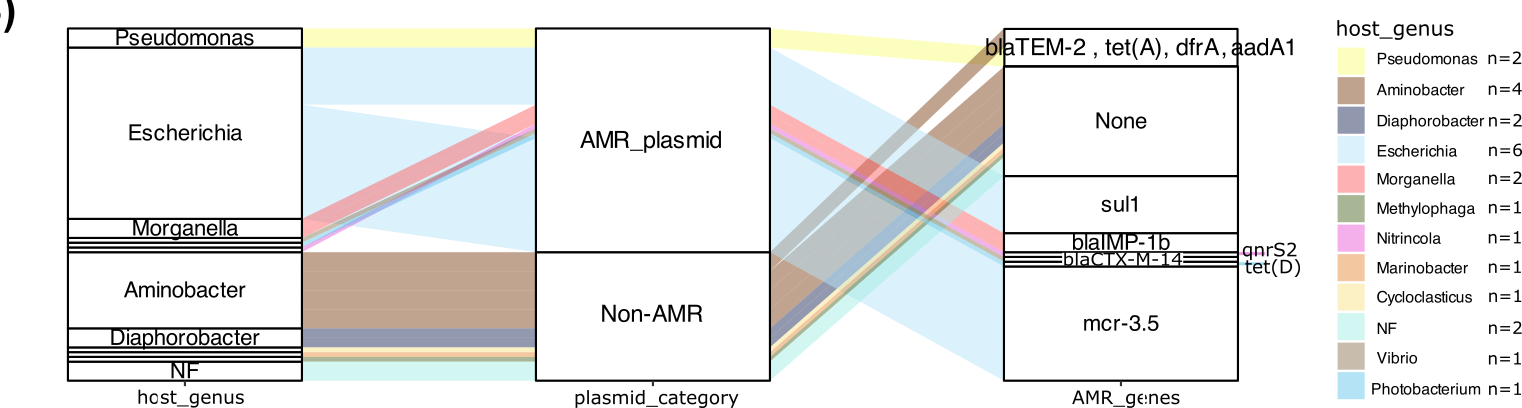


**Supplementary Figure 11. Distribution of plasmids carrying truncated TrbE variants.** Plasmid associations with host genera (colour-coded) and AMR genes.

| Term | DFn | DFd | F | p | p<.05 | ges |
| --- | --- | --- | --- | --- | --- | --- |
| Immigration | 1 | 8 | 88.66162 | 1.33E-05 | * | 0.791087 |
| Treatment | 1 | 8 | 80.0454 | 1.94E-05 | * | 0.773688 |
| Day | 1 | 8 | 2.325337 | 1.66E-01 |  | 0.160619 |
| Immigration:Treatment | 1 | 8 | 19.25207 | 2.32E-03 | * | 0.451226 |
| Immigration:Day | 1 | 8 | 10.49737 | 1.19E-02 | * | 0.463472 |
| Treatment:Day | 1 | 8 | 8.20471 | 2.10E-02 | * | 0.403046 |
| Immigration:Treatment:Day | 1 | 8 | 0.252774 | 6.29E-01 |  | 0.020377 |

Ezanova - error partitioned based on the repeated measures structure

| Effect | DFn | DFd | SSn | SSd | F | p | ges |
| --- | --- | --- | --- | --- | --- | --- | --- |
| Immigration | 1 | 8 | 1.04E+19 | 9.43E+17 | 88.66162 | 1.33E-05 | 0.791087 |
| Treatment | 1 | 8 | 9.43E+18 | 9.43E+17 | 80.0454 | 1.94E-05 | 0.773688 |
| Day | 1 | 8 | 5.28E+17 | 1.82E+18 | 2.325337 | 0.165791 | 0.160619 |
| Immigration:Treatment | 1 | 8 | 2.27E+18 | 9.43E+17 | 19.25207 | 0.002325 | 0.451226 |
| Immigration:Day | 1 | 8 | 2.38E+18 | 1.82E+18 | 10.49737 | 0.011878 | 0.463472 |
| Treatment:Day | 1 | 8 | 1.86E+18 | 1.82E+18 | 8.20471 | 0.021009 | 0.403046 |
| Immigration:Treatment:Day | 1 | 8 | 5.74E+16 | 1.82E+18 | 0.252774 | 0.628675 | 0.020377 |

**Supplementary Table 1. Determinants of plasmid density.** ANOVA (repeated measures) test to evaluate the impact of phage treatment and migration on the density of the RP4 plasmid.

| Effect | DFn | DFd | F | p | p<.05 | ges |
| --- | --- | --- | --- | --- | --- | --- |
| Immigration | 1 | 4 | 9.12E-01 | 3.94E-01 |  | 7.91E-02 |
| Day | 1 | 4 | 1.28E+04 | 3.67E-08 | * | 9.99E-01 |
| Immigration:Day | 1 | 4 | 7.28E-01 | 4.42E-01 |  | 0.101855 |

Ezanova - error partitioned based on the repeated measures structure

| Effect | DFn | DFd | SSn | SSd | F | p | p<.05 | ges |
| --- | --- | --- | --- | --- | --- | --- | --- | --- |
| Immigration | 1 | 4 | 1.19E+17 | 5.23E+17 | 0.911772 | 3.94E-01 |  | 0.079132 |
| Day | 1 | 4 | 2.76E+21 | 8.65E+17 | 12778.05 | 3.67E-08 | * | 0.999498 |
| Immigration:Day | 1 | 4 | 1.57E+17 | 8.65E+17 | 0.728112 | 0.44158 |  | 0.101855 |

**Supplementary Table 2: Determinants of phage density.** ANOVA (repeated measures) test to evaluate the impact of immigration on the density of the PRD1 phage.

Fixed Effects Estimates

| Term | Estimate (β) | Std. Error | df | t-value | P-value | Sig. |
| --- | --- | --- | --- | --- | --- | --- |
| (Intercept) | 32.764 | 5.608 | 25.41 | 5.842 | <0.001 | *** |
| Immigration (50%) | -32.9 | 7.931 | 25.41 | -4.148 | 0.0003 | *** |
| Day | -2.702 | 0.532 | 22 | -5.075 | <0.001 | *** |
| Immigration × Day | 3.266 | 0.753 | 22 | 4.337 | 0.0003 | *** |

Random Effects and Model Fit

| Group | Parameter | Variance | Std. Dev. |
| --- | --- | --- | --- |
| Replicate | Intercept | 7.605 | 2.758 |
| Residual | — | 8.504 | 2.916 |

**Supplementary Table 3. Statistical Analysis of PRD1 Density Trends across Days.** Statistical significance for phage recovery was assessed using a linear mixed model on log-transformed data, accounting for replicate as a random effect and utilizing Satterthwaite's approximation for degrees of freedom. The analysis reveals a highly significant Immigration × Day interaction (P=0.000265). This indicates that the temporal trajectory of phage density differs significantly, specifically from Day 8 onwards, and depending on whether immigration is present. Model: Linear Mixed Model (LMM) using Satterthwaite's method. Formula: log(mean+1)∼Immigration×Day+(1∣Replicate).

A. Primers for GFP Labeling of RP4

| Primer Name | Sequence (5′→3′) | Functional Components |
| --- | --- | --- |
| F1 | CCCCTTGGAGTAAGAACGC CCTGCAGG ATAAAAAACGCCCGGCGGCAACC | RP4 homology, SbfI site, GFP terminator |
| R1 | CAGCCTCCGCAGCTCGGGT TACGTA GGCGCGCCCCTCCTTGAC | RP4 homology, SnaBI site, GFP promoter |
| GFP Template | See access numbers sequence files from Supplementary data 2 | RP4:GFP sequence |

B. PCR Reaction Setup and Thermal Cycling

| PCR Master Mix | Volume (μL) | PCR Conditions | Temperature | Time |
| --- | --- | --- | --- | --- |
| 2 × PCR Mix (Dye Plus) | 10 | Initial Denaturation | 95∘C | 5 min |
| Primer F (10μM) | 1 | Denaturation | 95∘C | 1 min |
| Primer R (10μM) | 1 | Annealing | 60∘C | 30 s |
| ddH$_2$O | 6 | Extension | 72∘C | 1 min |
| GFP Gene Template | 2 | Final Extension | 72∘C | 10 min |
| Total Volume | 20 | 30–35 cycles |  |  |

C. Plasmid Digestion and Seamless Assembly

| RP4 Digestion (37∘C, Overnight) | Vol (μL) | Seamless Cloning Reaction | Vol (μL) |
| --- | --- | --- | --- |
| SnaBI Enzyme | 1 | Linearized RP4 Plasmid | 3 |
| SbfI Enzyme | 1 | GFP Amplicon | 2 |
| 10 × Buffer | 5 | 2 × Seamless Cloning Mix | 10 |
| RP4 DNA Template | 1 | H_2_O | 5 |
| H_2_O | Up to 10 | Total Volume | 20 |

**Supplementary table 4: Primers, sequences and cloning conditions used for fluorescence labeling of RP4 plasmid.**
